# Supplementary material for: PKAc is not required for the preerythrocytic stages of Plasmodium berghei
Source: Life Sci Alliance. 2019 May 29;2(3):e201900352. doi: 10.26508/lsa.201900352 (PMC6545604; doi:10.26508/lsa.201900352)
Supplement: Supplementary file 1 [file LSA-2019-00352_Supplemental_Data_1.doc]

Supplementary Information

Supplementary methods

STRING analysis

To predict interacting partners of PKAc, *P. berghei* PKAc protein sequence was submitted to STRING interaction database. Interaction diagram of *P. berghei* PKAc showed the interaction of PKAc with multiple proteins involved in various pathways. Phenotypes of those proteins were extracted from online databases like PlasmoGEM (https://plasmogem.sanger.ac.uk/phenotypes) and RMgmDB (<https://www.pberghei.eu/index.php>).
