# Supplementary material for: PKAc is not required for the preerythrocytic stages of Plasmodium berghei
Source: Life Sci Alliance. 2019 May 29;2(3):e201900352. doi: 10.26508/lsa.201900352 (PMC6545604; doi:10.26508/lsa.201900352)
Supplement: Supplementary file 2 [file LSA-2019-00352_TableS1.doc]

**Table S1. STRING analysis showing proteins with direct or indirect interaction with *P. berghei* PKAc along with their PlasmoGEM and RMgmDB phenotypes.**

| **PlasmoDB ID** | **Description** | **Phenotype** | **STRING interaction type** | **Association** |
| --- | --- | --- | --- | --- |
| PBANKA_143800 | cAMP-dependent protein kinase regulatory subunit, putative | NA | Experimental | (Varjosalo et al., 2013) |
| PBANKA_111530 | Ornithine aminotransferase | Essential | Predicted | Coexpression |
| PBANKA_133890 | Glideosome associated protein with multiple membrane spans 1, putative | Essential | Predicted | Coexpression |
| PBANKA_143760 | Glideosome-associated protein 45, putative | Essential | Predicted | Coexpression |
| PBANKA_071310 | Rhoptry neck protein 5, putative | Essential | Predicted | Coexpression |
| PBANKA_103210 | Rhoptry-associated protein 1 | Significantly slow growth | Predicted | Coexpression |
| PBANKA_100820 | cGMP-dependent protein kinase 1, beta isozyme, putative | Essential | Experimental | (Varjosalo et al., 2013) |
| PBANKA_113670 | Guanylate cyclase beta | Significantly slow growth | Experimental | (Cannon and Tatchell, 1987) |
| PBANKA_091030 | Guanylyl cyclase, putative | Significantly slow growth | Experimental | (Cannon and Tatchell, 1987) |
| PBANKA_090270 | Leucine rich repeat protein | Dispensable | Experimental | (Cannon and Tatchell, 1987) |

**References**

Cannon, J.F., and K. Tatchell. 1987. Characterization of Saccharomyces cerevisiae genes encoding subunits of cyclic AMP-dependent protein kinase. *Mol Cell Biol* 7:2653-2663.

Varjosalo, M., R. Sacco, A. Stukalov, A. van Drogen, M. Planyavsky, S. Hauri, R. Aebersold, K.L. Bennett, J. Colinge, M. Gstaiger, and G. Superti-Furga. 2013. Interlaboratory reproducibility of large-scale human protein-complex analysis by standardized AP-MS. *Nat Methods* 10:307-314.
