# Supplementary material for: PKAc is not required for the preerythrocytic stages of Plasmodium berghei
Source: Life Sci Alliance. 2019 May 29;2(3):e201900352. doi: 10.26508/lsa.201900352 (PMC6545604; doi:10.26508/lsa.201900352)
Supplement: Supplementary file 3 [file LSA-2019-00352_TableS2.doc]

**Table S2.** List of primers used in this study.

| **Primer Name** | **Primer Sequence 5’-3’** |
| --- | --- |
| **For real-time PCR** |  |
| 1307 | GTTGATCATGTATTTTCTGAAAG |
| 1308 | TCCAATTACAAATTCAAGAACCA |
| 1193 | CACGGCAAATTCAACGGCACAGT |
| 1194 | GACATACTCAGCACCGGCCTCA |
| 1195 | AAGCATTAAATAAAG |
| 1196 | GGAGATTGGTTTTGACGTTTATGTG |
| 1231 | AGAGAAGCAGCTGAAACAGC |
| 1232 | TCCCTTTAATAAATCATGGC |
| **For targeting construct^a^** |  |
| 1066 | ATCCGCGGGTTATGTTTGAAGGAAAAGATC |
| 1067 | CTGCGGCCGCTAAGCAAATATTGGATTATTATAT |
| 1068 | CTATTATCTAATACTGATGAATC |
| 1069 | ***TGTTTATTAAAATTA***CTACCAATCAAAAAATGGATC |
| 1070 | ATCTGCAGAAAGGAAAGTTTTGGTTATATTT |
| 1071 | GTGGTACCCATCTATACATATCCATCCAT |
| **For diagnostic PCR** |  |
| 1072 | GTCAACACATTAGACATATATG |
| 1216 | TATATAATTGAATAAATAACATAA |
| 1215 | GTTGTCTCTTCAATGATTCATAAATAG |
| 1073 | TTTTAAGGTGAAATGAATTTATG |
| 1409 | TTTATAACAGTTTGTACTAGTC |
| 1074 | GCAACTGGAGGTAAGTCTT |

^a^Restriction enzyme site underlined.
